# The acceptability and feasibility of a randomised trial exploring approaches to managing impacted fetal head during emergency caesarean section: a qualitative study

**DOI:** 10.1186/s12884-023-05444-5

**Published:** 2023-03-29

**Authors:** Gabriella Romano, Susan Ayers, Georgina Constantinou, Eleanor J. Mitchell, Rachel Plachcinski, Natalie Wakefield, Kate F. Walker

**Affiliations:** 1 Centre for Maternal and Child Health Research, School of Health Sciences, City, University of London, Northampton Square, London, EC1V 0HB UK; 2 Nottingham Clinical Trials Unit, University of Nottingham, Nottingham, UK; 3 National Childbirth Trust, Nottingham, UK; 4 Population and Lifespan Unit, School of Medicine, University of Nottingham, Nottingham, UK

**Keywords:** Impacted fetal head, Caesarean section, Second stage delivery, Obstetric complications, Randomised controlled trials, RCT

## Abstract

**Background:**

Caesarean sections (CS) account for 26% of all births in the UK, of which at least 5% are done at full dilatation, in the second stage of labour. Second stage CS may be complicated by the fetal head being deeply impacted in the maternal pelvis, requiring specialist skills to achieve a safe birth. Numerous techniques are used to manage impacted fetal head, however, there are no national clinical guidelines in the UK.

**Aim:**

To explore health professionals’ and women’s views on the acceptability and feasibility of a randomised controlled trial (RCT) designed to explore approaches to managing an impacted fetal head during emergency CS.

**Methods:**

Semi-structured interviews with 10 obstetricians and 16 women (6 pregnant and 10 who experienced an emergency second stage CS). Interviews were transcribed and analysed using systematic thematic analysis.

**Results:**

The findings considered the time at which you obtain consent, how and when information about the RCT is presented, and barriers and facilitators to recruiting health professionals and women into the RCT. Obstetricians emphasised the importance of training in the techniques, as well as the potential conflict between the RCT protocol and current site or individual practices. Women said they would trust health professionals’ to use the most appropriate technique and abandon the RCT protocol if necessary. Similarly, obstetricians raised the tension between the RCT protocol versus safety in reverting to what they knew under emergency situations. Both groups reflected on how this might affect the authenticity of the results. A range of important maternal, infant and clinical outcomes were raised by women and obstetricians. However, there were varying views on which of the two RCT designs presented to participants would be preferred. Most participants thought the RCT would be feasible and acceptable.

**Conclusions:**

This study suggests an RCT designed to evaluate different techniques for managing an impacted fetal head would be feasible and acceptable. However, it also identified a number of challenges that need to be considered when designing such an RCT. Results can be used to inform the design of RCTs in this area.

**Supplementary Information:**

The online version contains supplementary material available at 10.1186/s12884-023-05444-5.

## Introduction

Caesarean section (CS) accounts for 26% of all births in the UK, of which at least 5% are done at full dilatation in the second stage of labour, that is 34,000 births per annum [1]. There are a variety of reasons why a CS is conducted, some of which occur under emergency circumstances where there may be a threat to the woman or infant’s life. Emergency CS in the second stage of labour has greater maternal morbidity compared to emergency CS performed in the first stage of labour [2]. Second stage CS may also be complicated by the fetal head being deeply impacted in the maternal pelvis. This scenario occurs in 1.5% of all emergency CS [3] and the challenge for the maternity team is to disengage the head by hand when there is minimal space between the maternal pelvic bone and the deeply impacted fetal head. Complications that can arise for women and infants include: major haemorrhage; secondary to uterine or vaginal tears; longer delivery time; longer hospital stay; greater likelihood of bladder trauma; and injury to the infant such as bone fractures, hypoxic brain injury or occasionally death [2].

There is currently no clinical guidance on which techniques to use to manage an impacted fetal head during CS in the UK or US. There is a statement/opinion on impacted fetal head from both Canada and Australia/New Zealand [4, 5]. Many different techniques to assist in delivery of a deeply impacted head are reported, all of which aim to reduce the risk of maternal and fetal complications but the superiority of one technique over another is contentious. Most commonly used techniques include the reverse breech or ‘pull technique’, the ‘push technique’, and the fetal pillow [6]. The reverse breech or ‘pull technique’ is where an obstetrician delivers the baby during CS by gently pulling on the baby’s leg or legs to deliver the baby [7]. The ‘push technique’ technique is where a hand is inserted into the vagina to push the fetal head upwards whilst an obstetrician grasps the baby’s shoulders during CS to deliver the baby [7]. The fetal pillow® is a medical device that can be inserted into the vagina and inflated to push the baby’s head up in the pelvis without the need for a hand.

There are also no universal training programmes for maternity staff on how to manage an impacted fetal head and therefore little consensus on best practice. Reviews of the evidence also reach different conclusions, with one concluding the reverse breech extraction is associated with significantly lower maternal risks [8], and the other concluding the technique should be selected according to surgeon experience [7]. Further research is therefore needed to formalise the methods used by maternity staff during these critical incidents and to identify which are the most effective.

The MIDAS study (Managing an Impacted fetal heaD At emergency caesarean Section) (NIHR HTA 17/75/09) examined the acceptability and feasibility of different techniques of managing impacted fetal head. This programme included a national survey of health professionals about current practice [9]; interviews with women about the acceptability of different techniques [10]; a Delphi survey to get consensus on which techniques should be prioritised and tested in an RCT; and the design of two possible RCTs of different techniques for managing impacted fetal head during emergency CS [11]. Before proceeding to a definitive RCT it is important to explore whether this is feasible and acceptable and which of the two RCT designs is preferred.

The aim of this research was to qualitatively examine women’s and health professionals’ views on the acceptability and feasibility of an RCT to explore different approaches to managing an impacted fetal head during emergency CS, as well as views on the two RCT designs proposed. The results will provide the information needed to determine whether it is acceptable to conduct a future RCT in this area.

## Methods

### Design

A qualitative interview study of health professionals’ and women’s views on the acceptability and feasibility of RCTs designed to explore different approaches to managing an impacted fetal head at second stage CS.

### Ethical approval

Ethical approval was obtained from the West Midlands, Solihull, Research Ethics Committee (REC 19/WM/0118). The study was conducted in accordance with the ethical principles originating from the Declaration of Helsinki, 1996 [12]; the Principles of Good Clinical Practice and the UK Department of Health Policy Framework for Health and Social Care, 2017 [13].

### Sample

Three samples of participants were identified for the purposes of this study: obstetric doctors or trainees, women who had experienced emergency CS in the 24-months preceding the interviews; and primiparous women (either pregnant with their first child or had their first child in the previous 24 months). It was important to get the views of pregnant or primiparous women without experiences of impacted fetal head in order to get views from women who represent the population a future RCT would try to recruit. Women who experienced an emergency CS (and possibly impacted fetal head) may have different views because of their experiences.

#### Obstetricians

Obstetricians were recruited via social media and via mailing lists from a previous survey conducted as part of the MIDAS research programme [9]. Obstetricians were eligible if they were: National Health Service (NHS) staff working as an obstetrician or obstetric trainee on an obstetric unit, were 16 years or older; and were able to provide consent.

Twenty-three obstetricians expressed an interest in participating and 11 consented to take part. Of the 11 obstetricians who consented 10 were available to be interviewed on the dates available.

#### Women

Women were recruited via invitations sent from Nottingham University Hospitals NHS Trust and via social network channels (e.g. Twitter, LinkedIn and Facebook). Women were eligible if they had either (i) experienced a second stage CS in the 24 months preceding the date of interview; or (ii) were primiparous (either pregnant with their first child or had their first child in the previous 24 months); were aged 16 years or older (no upper age limit); had adequate spoken English; and were able to give informed consent. There were no exclusion criteria.

Twenty-six women expressed an interest in participating and 18 (69%) consented to take part. All 18 women were interviewed but audio files were corrupted for 2 interviews so these were not able to be transcribed.

### Procedure

#### Women

Women who had a second stage CS in the prior 24 months were identified from hospital records by a research midwife. Both urgency of CS (emergency versus elective) and indication for CS (failed instrumental delivery) are mandatory reporting fields on the maternity dataset, enabling eligible women to be identified. To try to ensure a representative sample, all women who were eligible over a 24 month period were identified from medical records. Of these, 80 were invited to participate: 43 who lived in deprived areas (i.e. an Index of Multiple Deprivation Decile (IMD) of 1 or 2) and 37 in other areas (IMD of 3–10).

Women who had a second stage CS were sent a letter of invitation and participant information sheet. Women were also invited via social network channels. Women who were interested in taking part returned a pre-paid postcard to the research team indicating their interest, providing contact details, and a signed consent form. Consent forms were signed and dated by the participant before they entered the study and checked by the research team who provided a countersignature upon receipt. The master files and documents were held by City, University of London in secure, locked facilities. At the end of the study documents were archived electronically and hard copies destroyed.

Pregnant and primiparous women were invited to take part through letters of invitation from the NHS teaching hospital and via social media networks (e.g. Twitter, LinkedIn, Facebook). Letters of invitation were sent to 80 pregnant women under the care of the NHS teaching hospital. Social media invitations used a digital poster which provided brief information about the study and contact details of the research team. Women who were interested in taking part were asked to contact the research team. All participants were sent a participant information sheet and consent form which they then returned to the research team.

**Fig. 1:**
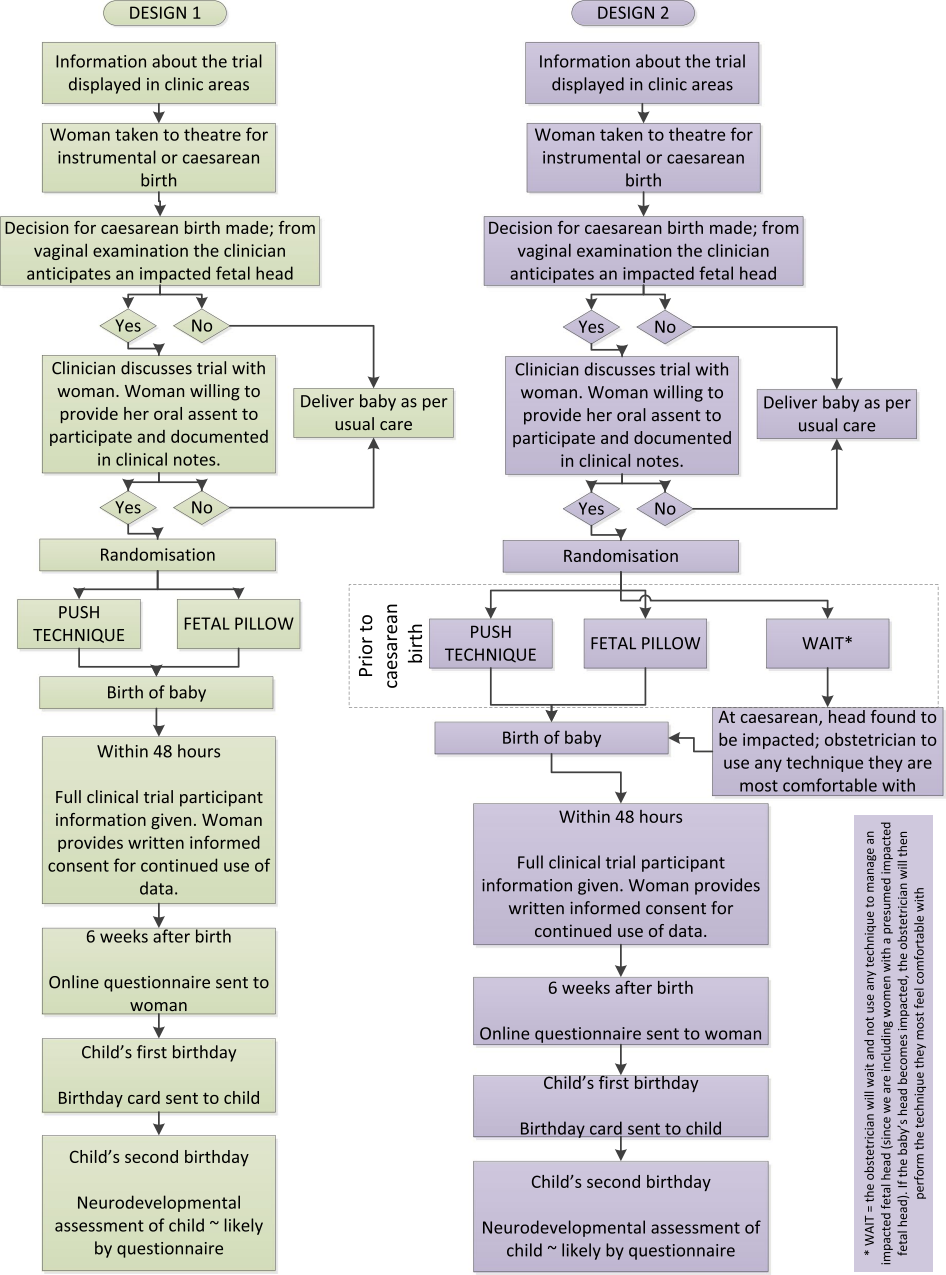
Proposed RCT designs

For women in both groups telephone or online interviews were arranged for a suitable time and conducted by a research psychologist experienced in conducting qualitative research. Interviews were conducted between October 2020 and February 2021. At the beginning of the interview, participants were asked to provide basic sociodemographic information such as age, ethnicity and relationship status. A semi-structured interview was then conducted which lasted approximately 45 min. The interview covered: (i) verbal descriptions of the push technique and fetal pillow; (ii) acceptability of different techniques; (iii) willingness to be involved in a trial of this type and (iv) a description of the two proposed trial designs using Fig. 1 as a visual aid, and women’s views on these designs. The visual aid was emailed to participants prior to telephone interviews or shared during online interviews so the researcher could describe the different designs to participants and answer any questions about them. The interview was conducted using the ethically-approved topic guide in Appendix A (see Supplementary Materials).

The interview was conducted by a researcher who was unaware of the participants’ childbirth details. If women wanted more information about their birth events and/or a referral to an obstetrician to find out more they were encouraged to contact their GP. The number of interviews conducted was dependent on women’s availability and data saturation. All interviews were audio recorded and transcribed.

#### Obstetricians

Obstetricians were recruited via social media or via mailing lists from a previous survey. Brief study information was shared via social media, along with contact details of the research team and how to contact the research team. The researcher responded to potential participants via email to send them a participant information sheet and consent form. Participants who returned a completed consent form were then offered a telephone interview. Interviews were conducted between October 2020 and February 2021 using a semi-structured interview schedule and lasted approximately 45 min. The interview covered: (i) acceptability of different techniques; (ii) acceptability and (iii) feasibility of a trial generally as well as the different trial designs (Fig. 1). The interview was conducted using the topic guide in Appendix B (see Supplementary Materials).

### Different trial designs

Two trial designs were developed based on previous work in the MIDAS study. This work included a national survey of health professionals about current practice [9]; interviews with women about the acceptability of different techniques [10]; and a Delphi survey to get consensus on which techniques should be prioritised and tested in an RCT [11]. The proposed trial designs are summarised in the visual guide in Fig. 1. The first is a randomised trial with two groups to compare the use of the push technique with the fetal pillow. Women would be randomised during emergency CS once it was established the fetal head was impacted. The second trial design has three groups which compare prophylactic use of the push technique or fetal pillow prior to CS, or waiting until during the CS when it can be established whether the fetal head is impacted or not. In the ‘wait’ group if the fetal head is impacted the obstetrician will use the technique they are most comfortable with. The difference between using these techniques prophylactically or as a treatment is that in design 2 the techniques are used before the CS and therefore before it is established whether the fetal head is impacted or not (prophylactic). In design 1 the techniques are used during CS when it is clear the fetal head is impacted (treatment).

The visual guide in Fig. 1 was used during interviews with participants to help them consider the different designs.

### Data analysis

Audio recordings were analysed using systematic thematic analysis [14, 15]. A combined inductive and deductive approach was used. Data were analysed using the following steps: first, all transcripts were read to become familiar with the data. Transcripts were then re-read and all initial codes identified and coded. When no further codes emerged (i.e. data saturation) all the codes were examined by two researchers (GR and SA, or GC and SA) who reached agreement on those that were most frequent or could be combined into main themes. Interviews for obstetricians and women were analysed separately by different researchers (GR and GC) and then results compared to identify main themes and subthemes from the different groups. The themes were relatively similar so are reported here together. It is noted in the results section where themes or subthemes arose from only obstetricians or only women. Finally, main themes were cross-checked against quotes to ensure quotes were reliably coded and represented the main themes. Analysis was facilitated by NVivo12, a specialist computer software package for qualitative analysis [16]. The approach used in this study was adapted from Ritchie and Lewis (2003) who described the three interrelated stages involved namely: (i) data management; (ii) descriptive accounts; and (iii) explanatory accounts [15].

## Results

### Sample characteristics

Sample characteristics for women are given in Table 1. Women were all from White British, Scottish or European backgrounds. Two thirds (69%) of women were educated to undergraduate or postgraduate degree level. The majority (88%) were married or living with their partner and in employment (96%). The average age was 32 (SD 4.9).

**Table 1:** Sample characteristics for women (*N* = 16)

**Characteristic**	N (%)
**Parity/birth group**	
Pregnant with first baby	5 (31)
Primiparous – gave birth in last 24 months	1 (6)
Emergency second stage CS	10 (63)
**Ethnicity**	
White British	14 (88)
White Scottish	1 (6)
White European	1 (6)
**Level of education**	
High school (GCSE)^a^	1 (6)
High school (A’ level / diploma)^b^	4 (25)
Undergraduate degree	6 (38)
Postgraduate degree	5 (31)
**Relationship status**	
Married	7 (44)
Living with partner	7 (44)
Single/not living with partner	2 (13)
**Employment**	
Employed	13 (81)
Self-employed	2 (13)
Unemployed	1 (6)
**Job Sector**	
Health, research and social care	7 (44)
Education	2 (13)
Customer services	2 (13)
Other	5 (31)
**Number of children**	
0—pregnant	5 (31)
1	9 (56)
2	2 (13)

^a^General Certificate of Secondary Education (age 16)

^b^General Certificate of Education Advanced Level (age 18)

Sample characteristics for obstetricians are given in Table 2. This shows an even gender balance, and that half the sample were consultants. Years since qualifying ranged from 3 to 27 with a mean of 18 years (SD 7.2). All participants had experience with impacted fetal head over the last 1 and/or 5 years. Exposure to cases of impacted fetal head in the previous year ranged from 0 to 20 with a mean of 7 cases (SD 6.4). Exposure over the previous 5 years ranged from 0 to 150 with a mean of 41 cases (SD 44.2).

**Table 2:** Sample characteristics for health professionals (*N* = 10)

**Characteristic**	N (%)
**Gender**	
Male	5 (50)
Female	5 (50)
**Grade of Qualification**	
Consultant	5 (50)
Specialty Registrar	3 (30)
Speciality Trainee year 6	1 (10)
Post Clinical Competency Training	1 (10)

### Thematic analysis

Analysis of the interviews with obstetricians and women identified four main areas for consideration: (I) Recruitment and Consent; (II) Feasibility and Acceptability; (III) Design Considerations; and (IV) Outcomes. The themes within each of these areas are shown in Table 3 and outlined in more detail below.

**Table 3:** Main areas for consideration and themes

Main Areas for Consideration	Themes
I: Recruitment and Consent	Tackling the timing of consent Information presentation Recruiting health professionals and women
II: Feasibility and Acceptability	Conflict between the trial and individual/site practice ^a^ Importance of training ^a^ Trust in health professionals’ judgement ^b^
III: Design Considerations	Which trial design is preferable Research protocols vs safety in what they knew ^a^ Authenticity of results When to randomise ^a^
IV: Outcomes	Outcomes relevant to obstetricians ^a^ Outcomes relevant to women ^b^

^a^Theme arose from obstetricians only

^b^Theme arose from women only

#### I: Recruitment and Consent

The first area for consideration identified a number of issues raised by obstetricians and women about barriers and facilitators to recruiting women and healthcare professionals into a trial. A number of ideas were shared on what to consider when engaging individuals in a trial. In relation to women’s recruitment, Tackling the timing of consent was thought to be critical and women and obstetricians frequently reflected on how best to present information to women in order to truly offer informed consent in an emergency situation.

It’s difficult because it’s already well known that taking consent for an emergency caesarean in itself isn’t full capacity consent giving because women in that situation aren’t able to remember or retain what they’ve been told. (HCP08).

I understand that you give the full clinical trial participant information forty-eight hours, erm, within a delivery but, you know, informed consent at that point it can be very difficult, especially if she hasn’t got an epidural. (HCP03).

Obstetricians were mindful of the need to consult women to identify the optimal way to approach to consent and randomisation.

These are extremely sort of tumultuary circumstances that someone finds them self in when they’re delivering their baby and I think how you do that sort of consent procedure and randomisation would be, would have to be very carefully studied with PPI [public and patient involvement] (HCP07).

Women also highlighted the barrier of trying to recruit under difficult circumstances when the woman is stressed and how this may influence the numbers of women willing to take part.

I think for most women in that situation, it's going to be quite a fraught time, and um, and a stressful time, and I'm guessing it would be that moment that we would be introducing the … the … the study and the trial. Um, and I think that could cause undue stress to the mum, having to make a decision as to whether, oh, I need to be doing this, and I worry that that might mean that your um, er, numbers or women that were prepared to take part, would be quite low. (Primiparous W017).

Information presentation was raised by women and obstetricians as really important when recruiting and consenting women. This included consideration over *when* to present women with information about the trial, *how much* information to present, the *content* of the information, and the *format* it should be presented in.

In terms of when to present women with information about the trial, views of obstetricians varied. Some thought women should be approached months before the birth and then, should an impacted head occur, at least women would already be aware of the trial when entering the labour ward. Others thought information should be given to women when they arrive at the labour ward.

I mean I think it would perhaps make more sense I think if all women in labour are actually given a leaflet about this so that they have time to think about it, just in case this were to happen to them. To give them more time to think about it. So any woman, I think, who is in labour and who is happy to take part in this trial, should be given this leaflet beforehand. (HCP06).

However, most women stated that they would rather be told about the trial taking place earlier to allow them to process information before being taken for a caesarean.

personally I feel like I would want to be approached before I went in theatre really ‘cos I feel like that’s, it's such an intense thing. You know, if, if they think a caesarean or emergency caesarean is looming, I would want someone to approach me before everything starts going, you know. The chance to, the midwife to give it to me and give me chance to just read it in my own time so I can process it, ask some questions about it, but then to be reminded of it, you know, before, before it goes too crazy. (ECS W02).

Women appreciated that impacted fetal head was rare, but would like to be given information about the trial in their antenatal midwife appointments to minimise the impact of being asked to give assent when in labour.

Having good information about it and probably quite early on as well so perhaps when putting together a birth plan or speaking through with my midwife plans rather than having a last minute decision. (Primiparous W15).

Of those women who stated a preference to be given information early, some pinpointed the 20^th^ to the 26^th^ week of pregnancy as an ideal time to approach them about a trial taking place in the hospital.

I think the earlier the better, so maybe at the point where they’re obviously, I wouldn’t say too early just in case there’s more complications with the pregnancy or whatever, but maybe around the, the twenty six week mark or kind of when they know what’s happening more with the pregnancy, so they’re over that worry, you know, of actually being pregnant and what’s happening and getting that out the way and they’re used to that. (ECSW010).

Being made aware earlier in pregnancy that a trial was taking place was also thought to improve the likelihood of women wanting to take part.

I think if I had the information about the trial before I gave birth, so then I’d be aware of it, that if an impacted head came up, oh this trial’s going to be discussed with me. I think if I knew about the trial before I went to give birth, then I would, I would be all for it, yeah, yeah. (ECS W013).

In terms of how much information should be given, obstetricians argued this was very dependent on the context where if you are asking women to consent in emergency situations then the information needs to be short and simple, whereas if you are providing information during pregnancy or after birth then more detailed information can be given.

Because if you've done an instrumental, and you have been unsuccessful, you've got about three or four minutes, so at that point you're going to say to them verbal, a verbal consent, but normally we would take a consent for a trial of instrumental in theatre. At that point, you should do the “do you want to join the trial, if this is unsuccessful?” (HCP02).

Women also reciprocated these views, explaining that the language needs to be as simple as possible due to the intensity of the situation.

Well it, it needs to be simple language, ‘cos you’re just kind of in a, in a space and in a zone where you’re not really understanding anything, and you, you’re that worried, because there is a complication and there is a problem. (ECS W013).

When the time comes that the information is presented to you erm, in a really, in layman’s terms and make sure that I’m completely clued up about what’s going to happen. (Primiparous W018).

Women stated that if information is being given at the time of a CS, this should be kept to a minimum and dense text avoided.

I don’t think I would be able to have ability to process so much you know, in the written format, or people talking at me and explaining the situation. (Primiparous W16)

In terms of the content of the information obstetricians emphasised the importance of outlining the problem the trial is trying to solve.

In the … in the information, it would be really useful to have some, not necessarily figures, but some kind of idea as to the nature of the problem we're trying to solve. So some kind of idea how frequent a problem occurs with a head that is difficult to deliver, either vaginally, or by a caesarean section. And that sometimes babies do have a lack of oxygen and we're trying to improve on that. Something like that, but in laymen's terms. (HCP02).

Women thought it would be useful to be given information about why the techniques had been chosen, including their effectiveness and safety as well as potential impacts on their baby and themselves. It was also deemed important to have a clear understanding of what will happen if she agrees to take part, particularly what will happen if the technique she was randomised to was not successful.

…be able to have some maybe facts or some science around why test this versus that and what is gonna happen to you and information on the safety of the baby and obviously if that technique doesn’t work how long is it tried for and then what happens, so just loads of information (ECS W003).

Women would also like to be reassured that either technique is appropriate and safe, and the obstetrician is well trained and confident in performing either technique.

I think I’d want to feel that being part of it… either option is great and the obstetrician would be equally as trained to do both, both of them are, well you can’t say both of them are as effective as the other cos you're trying to work that out aren't you whether they are, which ones the most effective? So I think, I guess it would be a reassurance that both of them are good (ECS W002).

In terms of the format of information, obstetricians discussed providing information in different forms (i.e. verbal, written, visual formats) and that the trial should be explained as clearly and simply as possible.

I would feel that a written version should be shown as well, so apart from verbal like a laminated simple version, so it would have to be very simple, it couldn’t have all the complexities that many studies do have, it would. (HCP08).

Women frequently discussed information being provided verbally, in written format and also using visual diagrams to help them understand the techniques. Women collectively felt that posters displayed in the clinics and waiting rooms were not as useful in providing information to women.

*I think maybe considering, I think if I was in the waiting area and I saw it, I’d probably read it, but the fact that I’d never expect it to happen to me. (ECS W010)*

*You said that the information was like available in antenatal clinics, but whether or not actually that’s enough, ‘cos I mean there’s loads of leaflets when you go to an antenatal clinic, you know, I mean I read them all because I get bored. But lots of women won’t, I think posters are quite passive. (Primiparous W001).*

It was thought that being actively given a leaflet to take away with them would be helpful as opposed to having information available in waiting rooms which they would probably not engage with.

do you kind of make sure they have the information, when the lady’s come for their scans is that something that you could, is there a leaflet that you can give them when they come to the scan or. I don’t know whether that would be beneficial to you, you know, because I mean I see leaflets on the table and I’ll be very honest, I didn’t really pick any up because I got kind of leaflets and paperwork from my midwife appointments that I didn’t pick anything extra up when I was at the hospital. (ECS W012).

Several women said they preferred to receive information verbally from their midwife or clinician, as a way to make them aware about the trial and also as a way to reinforce information provided separately or at a different time. It was discussed that this reminder could be provided when arriving for labour.

I think it should be a clinician who's involved in her care, So if she's under consultant lead care, then it can be the consultant, if they're under community midwife, then it can be the community midwife. And actually their part to play would be fairly small in … in that it's just making them aware this study's ongoing, here's the information sheet.(Primiparous W017).

So, if, you know, you do then go in, when you do go into labour and you know, the midwife who's looking after you might say oh, have you, you know, did you read the information about the research trial that you were given at your scan? So, if they say yes, obviously you know there's already some kind of understanding there and that kind of opens up the topic of conversation I suppose as to whether it is something they'd be happy to take part in if they needed to. (ECS W012).

Barriers and facilitators to recruiting health professionals and women were discussed by each group. Obstetricians reflected on how to engage other health professionals in a trial and recruitment in order to maximise participation. Advice included having a lead midwife for the trial to make sure midwives were engaged; and recruiting during the day when consultants are present.

You need to make some kind of provision for somebody to be the lead midwife on the trial, not the lead obstetrician, the lead midwife, because if you don't get midwives on board, it doesn't happen. (HCP02).

I think it would be feasible, I think recruitment would be better in daytime and when the consultants are around. (HCP03)

Variation between sites was clear. Obstetricians working in sites with no access to the fetal pillow saw this as an attractive reason to take part in the trial. In contrast, sites that were already using the fetal pillow saw this as less of an advantage to taking part.

I think it has to be really easy, has to be really er, clear, as to what is required. I think your er, possibly your jewel in the crown is that somebody is going to get the fetal pillow, so that's attractive. (HCP02).

In the last five years we’ve used the fetal pillow a lot more so the usual technique would be, well the current usual technique would be the fetal pillow. (HCP01)

Obstetricians also emphasised the importance of the research team being accessible in case issues arise.

I think the most important thing is making sure that the research team are as accessible as possible for any issues which can happen. Making sure that the documentation is sent in a timely basis. Erm, obviously all the documentation needs to be as simply written as possible. (HCP06).

Women expressed things that may influence recruitment to the trial these included concerns that introducing the trial early to primiparous women may cause them to worry about the birth of their baby or misunderstand their choice in taking part.

I suppose there is a little concern now as to, would that make them worry because most women are not planning for a caesarean birth, and so if you start talking about caesarean birth at that point, will they get confused and think oh gosh, they're going to force me into having a caesarean just for their study. (Primiparous WP17).

In addition, it was also discussed that knowledge of what to expect during birth may influence women’s willingness to take part, particularly for first-time mothers. It was discussed that a woman birthing their second child may view the trial as more acceptable.

maybe for not first-time parents because you’re only, you’ve already got no real idea, well I certainly don’t at the minute of what, what is going to go on. But if parents, if there’s a woman who’s had children before and has got a bit more experience in just the whole set up of being, being in that situation and they have more of an understanding then may, then I’d think it’s more acceptable… but the preference would maybe to have women who are more, who have had children in the past, maybe. (Primiparous W018).

Mm, I don’t know if it’s for my first child maybe not so much. I’ve probably been through the situation and know for next time then you’re a bit more clued up aren’t you and probably a bit more relaxed about things. (Primiparous W018).

It was also raised that primiparous women may be introduced to a concept of complicated birth through the study which they had not considered previously and this might cause them to worry.

So, then you’re having that discussion about, you’re introducing the idea of a traumatic birth, you know quite far you know in the ante-natal period. Yeah, I just, it’s really difficult. (ECS W006).

Women said they may perceive being asked to take part in a trial as meaning there is something wrong with their baby and were worried this would cause panic.

I think if I was being asked, you know, are you happy to take part in this I’d be, like, what's going on, like, is my baby still alive, like, it was such a panicked situation that I don’t know if that would make, that would kind of affect my decision making I guess. (ECS W002).

The word ‘trial’ was also stated by women to be negatively perceived as including ‘new’ or ‘poor’ techniques that might therefore impact on safety. Therefore, the importance of the language used to present women information about the trial was felt to be key to enhance recruitment.

I feel like people, they hear the word “trial”, maybe they’d be a little scared and put off, just because you know, you view trials as something that’s not certain. (ECS W010)

I think I’d just be a bit cautious of whether they would feel comfortable being used to test out these new techniques in case, you know, it didn't work or something went wrong. (ECS W012).

I think, as long as I had confidence that either technique was still potentially equally as successful as the other, you know, I wouldn’t want to think I was almost being a guinea pig for a technique which maybe was less um, less successful than the other one. (ECS W014).

#### II: Feasibility and acceptability.

The second area for consideration identified a number of issues raised by obstetricians and women about the acceptability and feasibility of conducting a trial into ways of managing impacted fetal head. A key theme raised by obstetricians was about being randomised to a technique that might cause conflict between the trial and individual/site practice for managing impacted fetal head, which might be a barrier to taking part in a trial. At an individual level, obstetricians pointed out that different individuals preferred or were familiar with different management techniques.

We're all … even though the procedure is similar but we're all completely different because we are influenced by previous outcomes, bad outcomes, by how many you have done. (HCP06).

I would need to be demonstrated how to use the fetal pillow because I haven’t done that. (HCP08)

I actually haven't had much training to be honest with you because we are so used to using the push technique here and I'm so used to using it. (HCP06)

Similarly, obstetricians mentioned that techniques required by the trial might conflict with existing site practices.

Getting us to revert to doing the push technique will require some bit of groundwork done just to convince our clinicians to also give it a bit of a trial so yeah … already we … we use the fetal pillow anyway. (HCP09).

So, I think for me, in my unit, I wouldn’t want to take part in the study… because my trainees are trained to anticipate an impacted head when they have done an unsuccessful forceps delivery. And to then ask them not to do those things. (HCP05).

However, obstetricians were aware that a trial is needed to reduce this variation in practice between obstetricians and sites in order to provide safer care.

I know that there is no recommended ideal and I also know that the way we improve quality in our service is to minimise variation and, therefore, having a trial proven best approach would potentially make for a safer obstetric care. (HCP08).

Given the variation between individuals and sites, obstetricians emphasised the importance of training in the different techniques prior to the trial so health professionals taking part were clear and confident in using them.

I would want to know what appropriate training consists of for fetal pillow, given the lack of any validated training, and if, if it’s within an actual research context, then yes. (HCP04).

Training will be, you said that, you know, maybe training developed, delivered by your team that will be great….Because that would have more credibility and give people confidence. (HCP03).

I mean I would suppose that the training would have to be done through a simulator to begin with, it's probably much easier. (HCP06)

Women were less explicitly aware of variation between individuals and sites but were concerned that obstetricians may be more experienced in, or favour, a particular technique. Women felt this would affect the obstetrician’s confidence and therefore influence women’s willingness to be a part of the trial.

I just want the tried and tested technique and it might be that this particular doctor's better at one than the other, or more experienced in one than the other (ECS W014)

If they're not confident then that's gonna be a major issue. Um, that's it, I think. (ECS W011)

Women also highlighted that it may not be acceptable to ask obstetricians to perform a technique they are not comfortable with.

I think the other, in terms of acceptability for me would be more is it, is it, is it acceptable to make an obstetrician do something that they don’t feel as comfortable with. (ECS W002).

Overall, women said they would trust health professionals’ judgement if impacted fetal head arose and would expect the team to do the most appropriate technique regardless of the trial. Women had confidence that the obstetrician would use their expertise to deliver the baby safely and would be happy to trust in their judgement.

any interventions during birth, I know that I’d actually be quite happy to, I would be pretty oblivious and would just go with whatever the doctor wanted to do… there’s not enough of a difference between the two that would strike me as wanting a preference but I don’t know if that’s down to my nature of I’ve always sort of said to myself I am quite happy to trust the team, the medical team’s judgement. (Primiparous W015).

Obviously the, the discretion of the Midwife or the Obstetrician who is, thinks it’s the best care for the child. But then ultimately if the OB says well this is the best thing for it then you have to listen to a professional opinion (Primiparous W018).

Several women discussed it being important to them to be made aware of what plans would be in place if the technique they were randomised to did not work to manage impacted fetal head. Specifically, their opinion of acceptability dependent on confidence that the obstetrician could move onto a different technique if needed.

I suppose I’d be comfortable if you said okay, we’ll try it for X period of time and then there’s plan B which we can move to quickly… then that would give me some confidence. (ECS W003).

Most women also considered the idea of a trial acceptable and valued it’s importance in improving care.

I think they’re an important part to you know, research and study and the only way we can improve is by trying things out.(ECS W010)

There’s no evidence one way or another that either technique is better, so you, we need that evidence. So, I think it’s, I feel like it’s acceptable, because we don’t know the right one, so you know, … we should find out (Primiparous W001).

#### III: Design considerations

This area for consideration consolidated reflections by obstetricians and women on the most appropriate trial design, and details of this kind of trial that might need to be considered.

Obstetricians had mixed views on which trial design is preferable. Six expressed a direct preference, with 2 preferring design one (2-arm trial of push technique versus fetal pillow) and 4 preferring design two (3-arm trial of prophylactic use of push technique, fetal pillow, and waiting). Obstetricians recognised that the two designs address slightly different questions.

If the question you’re asking is… ‘how do we prevent impacted fetal head?’ you want design two. If it’s ‘how do we deal with impacted fetal head?’ it’s design one. (HCP04)

Actually the two different arms do represent two different approaches, a prophylactic approach and a treatment approach, which would be [difficult to] compare in themselves… I think clinicians who thought like I did would be more likely to take part in a trial where they could actually diagnose… the condition rather than acting prophylactically. (HCP07).

Which design obstetricians preferred varied for several reasons. Those who preferred design one tended to do so because it was in keeping with their current practice and/or addressed the question directly (rather than prophylactically).

I mean yeah, personally I would say the design one is more comfortable for me because it's like clear and straightforward, which is I am, I've been doing now. (HCP10)

I wouldn’t be as keen on design two because I think that’s looking at prophylactics, how you reduce the impact of impacted fetal head, which … seems to be a different question of how do we deal with impacted fetal head and caesarean section. (HCP04).

Those who preferred design two said it was because it gave them more options in terms of clinical management and/or was in keeping with their current practice.

I'd rather go with the second one because you've got more options, so including more possible outcomes in your trial data. And if you think about it, the wait is you know, more in line with first do no harm, than the other two. (HCP02).

Women also had mixed views on which trial design is preferable. Fifteen expressed a direct preference, with 8 preferring design one (2-arm trial of push technique versus fetal pillow) and 7 preferring design two (3-arm trial of prophylactic use of push technique, fetal pillow, and waiting).Women’s preference for design one was chosen as it as many felt it would prevent the situation progressing to impacted fetal head.

The idea of waiting doesn’t fill me with confidence, because again if you’ve got a qualified person suspecting it, that is almost enough for me to go okay, well do something about it (laughs), and to start with the two techniques whereas waiting just feels like you’re increasing the risk of something horrible happening to the baby’s head. I don’t know, I might just be incorrect, but that’s sort of my feeling with it. (ECS W003).

If that was me, I think I’d go for the [design] one because you’ve got, you're sort of thinking ahead of time…So, to me, it’s like you're trying to prevent a problem from happening, rather than deal with the problem when it occurs. (ECS W011).

Whereas, design two was favoured by some women as it allowed for the inclusion of a wait arm which would rely on obstetrician discretion.

Yeah. I … I personally feel more comfortable with the second option, I think that my instinct is to trust the obstetrician's instinct, and with that being an option, that they then would continue with whichever technique they're most comfortable with. (Primiparous W017).

However, the concept of waiting caused much discussion with women, with worry that this would impact the safety of the baby and would waste time resolving the issue.

From what I am assuming, that [design two] could create more consequences, medical consequence and psychological consequences to the baby. And probably more traumatic, you know, experience for mum and the partner, you know, the partner being in the room, I’m assuming during the C-Section, probably would be pretty, pretty in, in distress too. So, you know, when, forget about the partner and the mum, thinking about the baby and the baby’s damage, probably for psychological and, and physical health, I’d, I’d rather not wait. (Primiparous W016).

Obstetricians reflected on the conflict between research protocols versus safety in what they knew. This is similar to the earlier theme of ‘Conflict between the trial and individual/site practice’ where obstetricians questioned whether they would stick to the protocol in emergency situations or revert to what they usually would do.

When you’re doing something surgically I think you have to really believe in what you’re doing. And so… what’s going to be a really important thing in your trial design is whether or not you allow people to deviate and if you do, do they then come off the trial completely or do you still say that in itself is kind of an interesting thing to measure? (HCP01).

Absolutely yeah, you want to do something … you want to rely on something at a critical time which you are most comfortable with isn't it, not something which you have hardly ever used. But then where it says like in the middle of the column, push technique and fetal pillow, have some sort of asterisk and say however if the clinician feels uncomfortable, or if the delivery is extremely difficult or whatever, they can switch to the other method they are more comfortable with, or something like that. (HCP06).

If I was very concerned that time was of the essence, to deliver a baby safely, I would be more concerned about doing a technique that isn’t my known best, efficient technique for me. (HCP08).

Obstetricians reflected on how this and other factors might affect the authenticity of results. Variation in how obstetricians carry out techniques and whether they deviate from the protocol would be really important in whether the results of a trial are robust and relevant to practice so these aspects need to be recorded as part of a trial.

What part of their wrist or arm or muscles are they using to do the pull, are they using the flexion of their wrist to create a pull, are they using the triceps by having an ergonomic straight arm, but all of these things will also affect your outcomes and so you will get some variability between practitioners. (HCP08).

Obstetricians thought another key issue for consideration was when to randomise and whether this is best to do before or during CS. A key concern was the time taken to randomise a woman in an emergency situation.

So my main worry about this was randomising a woman, the time taken to randomise and the discussion with the woman in the heat of the moment. (HCP03)

I’d want to know how you randomise, and making sure that that’s robust and all of your practitioners are truly comfortable using both, otherwise, you get skewing. (HCP04)

Because of the potential delay obstetricians thought it was preferable to randomise women before the CS and either know about this before going into the operating theatre or in the theatre as soon as they were aware the fetal head was impacted.

I think you’d need to go to theatre knowing what you were going to do. (HCP01)

So you're going to have to randomise before that, so that's going to tweak it a bit, because some of those won't have had an impacted head. Or you randomise … before you go into theatre, but then you don't open the answer until you've got the impacted head. But you don't want to delay. (HCP02).

Some obstetricians suggested consent to be in the trial should be taken at the same time as women consenting to an emergency CS.

We always consent them for Caesarean Section. So, our consent form has trial of forceps plus or minus Caesarean Section. So, it’s at that point that I think you should take consent and randomise them. (HCP05).

Women supported obstetrician views that in emergency situations they would want the obstetrician to abandon the trial and perform the technique they thought was best suited.

I mean you could have someone going down for a c-section and you going down design one, but then when you get in there within a minute you could think no, we just need to get this baby out now and then I suppose, you know, they are going to do what they feel most comfortable with if it's, if it's safer for the baby. Erm, if you’ve got the time then by all means it’s safe for mum and baby to take that little bit more time to pick which technique they're going to use then I think fine, as long as obviously the mums have of course consented to it, but if it is an emergency then, you know, it needs to be acted on there and then, then they just need to do what they need to do to get the baby out. (ECS W012).

Women also discussed their views on being randomised to one of the techniques, raising a concern as to whether or not the technique they were allocated to was suitable for them and would be the preferred technique in the obstetricians opinion.

I guess if it was me on the table, and some, somebody, well not somebody, the computer says “This procedure should be done”, I would think about, well you know, is this procedure most, most sensible for me at this time or did the clinician think about, you know, perhaps the other procedure would be more suitable for me, and for the person next door, it would be more suitable another procedure. But I think that there might be an unacceptable one for me, you know, or somebody else. (Primiparous W016).

#### IV: Outcomes

Both obstetricians and women suggested outcomes which might be relevant in a future trial. Results are shown in Table 4 for women and Table 5 for obstetricians. Outcomes important to women were mostly about the health and safety of the women, infant, and her and her partner’s experience. In contrast, obstetrician’s generated a lot more detailed clinical outcomes for women and the infant, as well as staff outcomes. There was very little overlap between the outcomes mentioned by women and obstetricians, with the exception of safety of the mother and baby, and women’s experiences. However, many of the outcomes specified by obstetricians were consistent with women’s concerns for maternal/infant safety and wellbeing.

**Table 4:** Important outcomes for women (*N* = 16)

Category	Measure	N (%)
Infant outcomes	Time taken to resolve impacted fetal head	8 (50)
	Long-term disability/Impact on QoL	2 (12.5)
	Psychological/Physical Trauma	2 (12.5)
	Infant stress	2 (12.5)
	Infant death	2 (12.5)
	Developmental outcomes	1 (6.25)
Maternal outcomes	Women’s/partner’s experiences of birth	7 (43.75)
	Invasiveness	4 (25)
	Stress	2 (12.5)
	Increased time in recovery	2 (12.5)
	Internal Damage or Tearing	1 (6.25)
	Excessive Pain	1 (6.25)
Clinical staff	Experience/views on performing techniques	3 (18.75)

**Table 5:** Important outcomes for obstetricians (*N* = 10)

Category	Measure	N (%)
Infant outcomes	Fetal Trauma/ Damage to the baby	5 (50)
	Safety of the baby	4 (40)
	NICU for 48 h/Special care	4 (40)
	Neonatal Mortality	3 (30)
	Fractured Skull	3 (30)
	Ease of delivery of baby’s head	2 (20)
	Scalp Injury/Bruising	2 (20)
	Hypoxia	2 (20)
	Cord gases	2 (20)
	APGAR score	2 (20)
	PHs	1 (10)
	Bleeding	1 (10)
	Acidosis	1 (10)
	Ventilatory support	1 (10)
Maternal outcomes	Blood loss/postpartum haemorrhage	8 (80)
	Uterine tear	3 (30)
	Extension to the uterine incision	2 (20)
	Speed of recovery and discharge	2 (20)
	Mother’s experience	2 (20)
	Safety of the mother	1 (10)
	Physical trauma to other structures	1 (10)
	Mother needing surgical repair	1 (10)
	Atonia	1 (10)
	Tying off ureters	1 (10)
	Angle tears	1 (10)
	Hysterectomy	1 (10)
	Infection	1 (10)
Clinical staff	How comfortable staff feel to use technique	2 (10)
	Stress on Staff	1 (10)
	How difficult it is to teach	1 (10)
Clinical outcomes	Surgical/operating time	4 (40)
	Cost	2 (20)
Methodological confounders	Who deviates from the protocol and why	1 (10)

## Discussion

This study aimed to explore women’s and obstetricians’ views on the acceptability and feasibility of an RCT examining different approaches for managing an impacted fetal head during emergency CS, as well as views on the two RCT designs proposed. Results found women and obstetricians thought a trial was important and most thought it would be acceptable and feasible. However, they raised a number of issues for consideration under four areas of *Recruitment and consent, Feasibility and acceptability, Design considerations* and *Outcomes,* which are discussed in turn.

Recruitment and consent considered the issue of when consent is obtained and the difficulty of trying to do it under emergency situations. Women thought a good time to provide information about the RCT was in the second trimester when more detailed information could be given and they had time to ask questions and consider it fully. Women and obstetricians also raised the importance of the content and format of information being tailored to the circumstances under which it is given. These findings are consistent with previous literature [10, 17]. The difficulty of obtaining informed consent in emergency situations has been widely debated. The advantages and disadvantages of obtaining consent in pregnancy are recognised by guidelines for perinatal research [18], which acknowledge that informing women of possible obstetric complications might create unnecessary anxiety during pregnancy, particularly if the complication is rare [18]. This concern was echoed by a few women in this study. On the other hand, trying to obtain consent during emergency situations may delay life-saving treatment [19] and women may be unable to give fully informed consent [20].

Proposals for gaining consent in emergency circumstances therefore include deferred consent where women are randomised prior to the procedure and consent is asked for afterwards [21]; or a two-stage process of obtaining brief verbal consent prior to the procedure followed by written consent after the event [22]. Qualitative studies of women’s experiences of providing written consent in early labour or using the two-stage process in critical situations suggest women’s experiences are broadly similar and all women believed they were given enough information to make a decision despite differences in the timing and amount of information given [17]. Other studies suggest women’s decisions are more influenced by the quality of interactions with healthcare professionals and concern for the safety of their baby, rather than the timing or amount of information given [21, 23].

The area of feasibility and acceptability included potential barriers and facilitators to getting health professionals and women to conduct or take part in the RCT. Barriers were predominantly the conflict between the preferred techniques or practices of obstetricians and obstetric units and the RCT protocol. Facilitators were the attractiveness of being provided with fetal pillows (in units where they did not have them), good training in the techniques included in the RCT, and the ability to over-ride the RCT protocol in critical situations where clinical judgement and safety warranted it. Women also said they would trust health professionals to use the most appropriate technique and abandon the RCT protocol if necessary. The importance of good training in trial procedures/techniques is well recognised [24]. However, the finding that both obstetricians and women would want the option to override the RCT protocol makes clinical trial conduct challenging. Establishing whether recruiting obstetricians were in equipoise prior to participation would be crucial [25] and ensuring adherence to the randomised allocation would also be important. The findings of this study will influence the study design of a randomised trial in this area.

Design considerations showed there were varying views on which of the two RCT designs would be preferred – and advantages and disadvantages to each. There are two potential timings of the intervention: 1) using prophylactic or early dis-impaction prior to starting the CS when the vaginal examination findings suggest there may be an impacted fetal head 2) ‘treatment’ or late dis-impaction after delivery has been attempted at CS. The choice of design depends on whether the aim is to evaluate the effectiveness of techniques to manage impacted fetal head before or during CS. One technique for managing an impacted fetal head is the fetal pillow which takes 60s to insert. This may make it less appropriate to use for ‘treatment’, as it would delay the uterine incision to delivery interval, although previous research suggests it results in similar [26, 27] or slightly better outcomes [27, 28]. This technique can only be fairly compared with other techniques if used at the same time.

A range of important maternal, infant and clinical outcomes were raised by women and obstetricians. These were slightly different in focus, with women focusing on wellbeing and safety of themselves and the infant, as well as the experience. Outcomes mentioned by obstetricians were more clinically focused and specific but most were consistent with women’s concerns. Choice of outcomes for the trial will be guided by this work and a Delphi process and expert consensus meeting which was undertaken to reach consensus on which outcomes should be chosen for a trial.

### Methodological limitations

This study has a number of limitations that should be considered before drawing conclusions. The sample of women was heterogenous: all women were white and two thirds were educated to degree level or above, despite our attempts to recruit a diverse sample of women. Similarly, the health professionals interviewed were all obstetricians. More research is therefore needed to determine the views of women from ethnic minority groups and other maternity care professionals, such as midwives, who help manage impacted fetal head. A second limitation is the challenge of presenting two different, complex RCT designs to participants. Although participants had a good understanding of the different techniques, some of the women found the trial designs hard to understand, especially if they had a limited understanding of research methods and procedures. To address this we used a visual diagram (Fig. 1) outlining the two designs so these could be explained and talked through with each participant before asking for their views on the proposed trials.

## Conclusion

This study is the first to examine women and obstetricians views on specific RCT designs to evaluate different techniques for managing impacted fetal head. This study provides a greater understanding of the complexities involved in conducting research both in this area and more widely when conducting research involving obstetric emergencies. As such, it will directly inform future RCTs in this area. This research suggests an RCT designed to evaluate different techniques for managing an impacted fetal head would be feasible and acceptable but identifies a range of considerations that are important when designing such an RCT. Results can be used to inform the design and conduct of a definitive RCT in this area.

## Supplementary Information

**Additional file 1.**

## Data Availability

The datasets used and/or analysed during the current study available from the corresponding author on reasonable request.

## Abbreviations

CS: Caesarean Section
IMD: Index of Multiple Deprivation
MIDAS: Management of an ImpacteD fetal heAd during emergency caesarean Section
NHS: National Health Service
NIHR HTA: National Institute of Health Research, Health Technology and Assessment funding
RCT: Randomised Controlled Trial
REC: Research Ethics Committee
SD: Standard Deviation
UK: United Kingdom
