# Supplementary material for: The acceptability and feasibility of a randomised trial exploring approaches to managing impacted fetal head during emergency caesarean section: a qualitative study
Source: BMC Pregnancy Childbirth. 2023 Mar 29;23:216. doi: 10.1186/s12884-023-05444-5 (PMC10052859; doi:10.1186/s12884-023-05444-5)
Supplement: Supplementary file 1 — Additional file 1. [file 12884_2023_5444_MOESM1_ESM.docx]

**APPENDIX A | INTERVIEW GUIDE FOR WOMEN**

**AIM OF INTERVIEWS:**  To conduct individual telephone or video interviews with women who have experienced a second stage CS and primiparous women to determine the acceptability and feasibility of the planned RCT.

**STRUCTURE**

1.0 Introductions

2.0 Confidentiality and Consent

3.0 Sociodemographic Information

4.0 Interview

5.0 Information about additional support if needed after the session

**1.0 INTRODUCTIONS**

We are interested in women’s views of the acceptability and feasibility of clinical study we have designed to test several different techniques to manage an ‘impacted fetal head’ during labour. We are interested in the views of women who have experienced a late caesarean (second stage caesarean) and also the views of women who are pregnant for the first time to see whether this kind of trial would be acceptable and possible to do in the future.

Caesareans performed late in labour, where the neck of the womb is fully open and the baby’s head has entered the pelvis can be complicated. If the baby’s head is deeply wedged in the woman’s pelvis it can be difficult to lift it up so the baby is delivered.  This is called ‘impacted fetal head’. There are a number of different techniques that can be used when the baby’s head is impacted, however, it is not clear which is the best or most effective. This project is looking at whether it would be possible to do a clinical research trial to test these different techniques and find out which is most effective.

**2.0 CONFIDENTIALITY & CONSENT**

- Please can you confirm that you have read and understood the information sheet for this study?
- As you’ll know from the information sheet your participation is voluntary and that you are free to withdraw at any time, without giving any reason. The interview will be recorded and the transcript will be anonymised to maintain your confidentiality.
- Re-confirm consent (verbally): Are you still happy to give your consent to be interviewed?

**3.0 SOCIODEMOGRAPHIC INFORMATION**

- What is your date of birth?
- What is your ethnicity?
- When was your most recent birth?
- How many children do you have?
- Can you confirm your relationship status? (Single, Married, Divorced, etc.)
- What is your highest level of education? (Diploma, Graduate (BA, BSc), Post Graduate (MSc, PhD, etc.)
- What is your current working status? (Unemployed, Benefits, Full Time, Part-Time, Freelance, Contract etc.)
- What industry do you work in? (e.g. Retail, Catering, Health, Academia, Hospitality, etc.)
- What is your occupation?

**4.0 INTERVIEW**

**Different types of techniques**

Show women the different types of techniques chosen for the study ([insert names of techniques])

1. What are your views on these different techniques for managing impacted fetal head?
2. If you were in this situation, which technique(s) would you prefer to be used with you and your baby? **Probes**: Why do you prefer this option? If no preference, why?
3. What would you see as the advantages of the technique you prefer?
4. What would you see as the disadvantages?
5. What would be the most important outcome(s) for you in terms of which technique is best to use? **Probes:** Time to deliver, woman not requiring critical care, no injury caused to the woman, no complications to the baby, need for neonatal support reduced
6. Do you think any of these technique(s) would **not** be acceptable to parents?

**Willingness to be involved in a trial**

 Explain what an RCT is. To test the effectiveness of these techniques, we would need to randomly allocate women to have one particular technique used on them and their baby if the baby’s head becomes impacted during birth and a caesarean is necessary.

1. What are your views of how **acceptable** it would be to do this research with women during the birth of their baby?
2. If asked to, would you be prepared to take part in a trial looking at the most effective technique to manage impacted fetal head?

Probes**:** Yes, please can you explain why? No or not sure, please can you elaborate on why?

1. What factors are most important to you in deciding whether to take part?
2. What would encourage you to take part in the trial?
3. What would put you off taking part in the trial?
4. What could the research team or clinicians do to change this?

**Trial design**

In terms of being invited to take part in the trial: we have a plan for how and when we will approach women about the study.  Brief description of the 2 trial designs and patient pathway through these trials:

8a Do you agree with our plan for when women will be approached and asked to take part?

8b Would you recommend a different time to approach women?

8c How would you like to be approached?

Probes: Who? How (e.g. in person, writing)

8d What would you like to know when being invited to take part?

8e What are your views on tour plan for obtaining consent from women to take part?

Probes**:** Timing, who, how?

1. What advice would you give a team of clinicians and researchers about how to carry out this research trial in a way that is most **acceptable** to women and their babies?
2. What **outcomes** do you think research should look at when considering how good different techniques are?
3. The main outcome for women we have chosen for the study is [insert maternal primary outcome] do you think this is an important outcome for the study to examine?
4. The main outcome for the baby we have chosen for the study is [insert neonatal primary outcome], do you think this is an important outcome for the study to examine?
5. Is there anything else you would like to add?

**5.0 INFORMATION ABOUT ADDITIONAL SUPPORT IF NEEDED**

**APPENDIX B | INTERVIEW GUIDE FOR HEALTH PROFESSIONALS**

**AIM OF INTERVIEWS:**  To conduct individual telephone or video interviews with obstetricians/senior trainee obstetricians to determine the acceptability and feasibility of the planned RCT.

**STRUCTURE**

1.0 Introductions

2.0 Confidentiality and Consent

3.0 Sociodemographic Information

4.0 Interview

**1.0 INTRODUCTIONS**

 We are interested in your views of the acceptability and feasibility of a randomised controlled trial we have designed to test which of the different techniques to manage ‘impacted fetal head’ during a late caesarean section are best.

Caesareans performed late in labour, where the neck of the womb is fully open and the baby’s head has entered the pelvis can be complicated. If the baby’s head is deeply wedged in the woman’s pelvis it can be difficult to lift it up, so delivery can occur.  This is called ‘impacted fetal head’. There are a number of different techniques that can be used when the baby’s head is impacted, however, it is not clear which is the best or most effective. This project is looking at whether it would be possible to do a clinical trial to test these different techniques and find out which is most effective. We are interested in the views of health care professionals to determine whether the clinical trial we have designed is acceptable to them.

**2.0 CONFIDENTIALITY & CONSENT**

- Please can you confirm that you have read and understood the information sheet for this study?
- As you’ll know from the information sheet your participation is voluntary and that you are free to withdraw at any time, without giving any reason. The interview will be recorded and the transcript will be anonymised to maintain your confidentiality.
- Re-confirm consent (verbally): Are you still happy to give your consent to be interviewed?

**3.0 SOCIODEMOGRAPHIC INFORMATION**

- What is your health profession or discipline?
- What is your grade of qualification? e.g. ST3-5, ST6-7, Consultant, SAS doctor etc
- How many years have you been practicing since qualification?
- How would you rate your exposure to impacted fetal head in your role? e.g. approx. how many times in the last year / 5 years

**4.0 INTERVIEW**

**Different types of techniques**

Outline the different techniques chosen for the randomised controlled trial [push technique or fetal pillow] What technique would you usually use when faced with this scenario?

**Probes:**  Why this technique (or combination of techniques)? What would be your next technique if this wasn’t successful? Pros and Cons to this method? Have you always used this technique?

What would be the most important outcome(s) for you in terms of which type of technique is most effective? **Probes:** Time to deliver, woman not requiring critical care, no injury caused to the woman, no complications to the baby, need for neonatal support reduced.

 Would you be willing to use [push technique or fetal pillow] within the context of a randomised controlled trial if appropriate training were provided to you?

**Acceptability of a trial**

Thinking about the randomised controlled trial of techniques for managing impacted fetal head we have designed, what are your views on how **acceptable** it is to conduct research like this? Would you be prepared to take part in this trial testing the effectiveness of different techniques to manage impacted fetal head?

**Probes**: yes, why? no, why? Not sure, why?

What factors are important to you in deciding whether to take part?

**Probes**: types of techniques trialled; resources; training; timing

What advice would you give the research team about how to make the trial more **acceptable** to healthcare professionals?

**Feasibility of a trial**

The trial we are proposing to do will compare push technique with the fetal pillow.

How **feasible** do you think it would be to run this trial in your Trust?

What do you think would be the main barriers or challenges in your Trust to running this trial? What could be done to facilitate the trial running at your Trust?

What advice would you give the research team about how to make the trial more **feasible** to implement in different Trusts? Is there anything else you would like to add?
